# Role of jasmonic acid and salicylic acid in salinity stress mitigation in plants

**DOI:** 10.3389/fpls.2026.1824082

**Published:** 2026-04-24

**Authors:** Pravej Alam, Fadime Karabulut, Javed Iqbal, Renuka Sharma, Amin Fathi, Eyüp Bagci, Mehmet Firat Baran, Thamer Albalawi, Mohammad Faizan

**Affiliations:** 1 Department of Biology, College of Science and Humanities, Prince Sattam Bin Abdulaziz University, Alkharj, Saudi Arabia; 2 Hizan Vocational School, Bitlis Eren University, Bitlis, Türkiye; 3 Department of Botany, Bacha Khan University, Charsadda, Khyber Pakhtunkhwa, Pakistan; 4 Shree Guru Gobind Singh Tricentenary University, Gurugram, Haryana, India; 5 Department of Agronomy, Islamic Azad University, Amol, Iran; 6 Department of Biology, Faculty of Science, Firat University, Elazıg, Türkiye; 7 Departments of Biosystems Engineering, Faculty of Agriculture, Siirt University, Siirt, Türkiye; 8 Botany Section, School of Sciences, Maulana Azad National Urdu University, Hyderabad, India

**Keywords:** hormonal crosstalk, jasmonic acid, phytohormones, ROS detoxification, salicylic acid, salinity stress, toxic effect

## Abstract

Salinity stress is exacerbating across the world, causing physiological, molecular, and biochemical changes in plants. These phenomena can significantly deteriorate crop yield and quality. Phytohormones play vital role to the mitigate environmental stresses that negatively affect plants and help them to tolerate harsh conditions. Of these phytohormones, derivatives of salicylic acid (SA) and jasmonic acid (JA) are especially important because they take part in several signal transduction pathways that regulate a wide range of physiological and molecular processes in plants. JA lowers the oxidative stress caused by an increase in hydrogen peroxide in salt-stressed plants by activating the enzymes superoxide dismutase and ascorbate peroxidase, which are linked to higher levels of α-tocopherol, phenolics, and flavonoids. Phenylalanine ammonia-lyase activity is induced, which explains the rise in phenolics and flavonoids. When plants under salt stress are treated with exogenous SA, the growth inhibition is lowered. Carotenoid content significantly increases as a result of SA-induced antioxidant system activation, but H_2_O_2_ concentration, lipid peroxidation, and ion leakage significantly decrease. Consequently, this review offers a clearer picture of the function of JA and SA and shows the potential applications of phytohormonal additives in plant enhancements, such as engineering research to increase plant resistance to salinity stress.

## 1 Introduction

Soil salinisation has emerged as one of the most significant environmental constraints limiting crop growth and productivity in the modern era. Elevated salinity reduces the water potential of the soil solution, promotes excessive accumulation of reactive oxygen species (ROS), and disrupts ionic balance, leading to ion toxicity and multiple physiological and biochemical dysfunctions in plants. In many crops, saline irrigation water adversely affects seed germination, vegetative growth, and yield, particularly in arid and semi-arid regions (Bakry et al., 2024). Salinity is also known to induce oxidative stress, physiological drought, and nutrient imbalances, ultimately impairing plant morphology and productivity due to ionic disequilibrium and toxicity (Zörb et al., 2019). To cope with salt stress, plants activate defense mechanisms that regulate ROS levels, maintain physiological and biochemical processes, and sustain growth and productivity. These responses help mitigate ionic toxicity, prevent nutrient deficiencies, protect enzyme activity, and reduce the risk of cellular damage and death (Fernandes et al., 2022). Additionally, plants have evolved complex molecular and biological defense systems, including phytohormone-mediated signaling pathways that enhance tolerance under saline conditions. Salinity can originate from both natural and anthropogenic sources. It is often caused by poor irrigation practices, inadequate drainage, absence of salt-tolerant crops, and unsustainable agricultural management. Environmental factors such as climate change and prolonged drought further aggravate salinity issues (Kohler et al., 2010). While natural processes remain the primary contributors to soil salinity, human-induced factors such as excessive land use and improper irrigation significantly accelerate its development (Mahmuduzzaman et al., 2014). Soil salinity is also influenced by parent materials, including volcanic rocks, sandstones, lagoon sediments, and basaltic formations, as well as by hydrological and climatic conditions. High evapotranspiration rates in arid and desert regions intensify salt accumulation and sodicity. Improper irrigation systems promote the accumulation of soluble salts in the root zone, and if these salts are not effectively leached with good-quality water, they progressively reduce soil fertility and crop productivity. It is estimated that approximately 45–50% of irrigated lands, especially in lowland and arid regions, are affected by soil salinity, posing a serious threat to global agricultural sustainability.Top of Form.

## 2 Bottom of form

Assessing the importance of salinity for future agricultural production has become more difficult due to several recent attempts to quantify the degree of human-induced secondary salinisation (Hossain, 2019). Approximately 20% of arable land and 50% of irrigated land are affected by salinity globally (Birati et al., 2025). 50 mM NaCl can result in a yield loss of over 70% of the product, making salinity stress a significant crop production constraint (Hasanuzzaman et al., 2013). Physiological drought resulting from salt stress leads to decreased crop growth and yield, delayed seed germination, and delayed establishment of seedlings.

Alternative methods for resolving this issue include salt-tolerant cultivars and seed treatments with nutrients and plant growth regulators (PGRs) (Tarchoun et al., 2022). Despite being tiny molecules used in small doses, plant growth regulators—which affect cell division, elongation, and regeneration—have a major effect on plant growth *in vitro* (Pasternak and Steinmacher, 2024). Gibberellins, ethylene, abscisic acid, auxins, cytokinins, and other plant growth regulators may be discussed. Auxins and cytokinins, as well as their ratio, are important PGRs for organogenesis and somatic embryogenesis (SE) processes. The plants themselves produce natural PGRs. They interact with the additional synthetic or natural plant growth regulators to promote development *in vitro* (Pasternak and Steinmacher, 2024). In addition to directly influencing plant cellular mechanisms, a range of exogenously applied regulators may modify the synthesis, destruction, activation, transport, or specificity of endogenous hormones or other PGRs (Karabulut, 2024). PGRs applied externally maximise the physical metabolic conditions for seed germination in situations where environmental stress affects endogenous hormones. High salt concentrations have the potential to inhibit natural hormones, so PGR-soaked seeds supply enough hormones for healthy growth (El Sabagh et al., 2022). PGRs have improved seed performance, nutrient reserves, and root proliferation and yield under stress for a variety of vegetable crops by increasing physiological activities during pre-soaking, priming, and nutrient applications (Garai and Patra, 2024). Rice seedlings under salt stress recover better when JA is applied externally. According to Kang et al. (2005), plants of salt-sensitive cultivars seem to have less JA than plants of salt-resistant cultivars. When tomato and barley plants are under salt stress, external administration of SA improves photosynthesis, shoot growth, and survival rate (Li et al., 2025). Studies have revealed that salt stress affects about 20% of agricultural land, or 50 million hectares (Shrivastava and Kumar, 2015). By decreasing water absorption or altering solute potential, salt stress affects the osmotic potential of plants, which in turn impacts hydraulic conductivity. This can hinder plant growth and ultimately cause cell death by causing ionic imbalance, harm to the mitochondria and chloroplasts, and disruptions of water relations and photosynthesis (Sheteiwy et al., 2018). To scavenge ROS and stop excessive ROS accumulation and oxidative damage, plant cells complexly synthesise ascorbate peroxidase (APX), catalase (CAT), and superoxide dismutase (SOD) (Ulhassan et al., 2019; Sheteiwy et al., 2021). Furthermore, it is decreased by non-enzymatic antioxidants like proline (Ahmad et al., 2016), phytohormones like ethylene and abscisic acid (ABA), and increased concentrations of secondary metabolites like flavonoids and total phenols (Mir et al., 2018; Garcia de la Garma et al., 2015).

Salinity induces numerous morphological, molecular, physiological, and biochemical changes in plants. Initially, salt stress leads to osmotic stress due to reduced water potential caused by dissolved salts, followed by ion toxicity from excessive salt accumulation and oxidative stress resulting from the generation of reactive oxygen species (ROS) that disrupt cellular functions (Zhang et al., 2018; Botella et al., 2007). Plants employ several adaptive mechanisms to cope with salinity, including maintenance of ion homeostasis, ion exclusion and compartmentalization, ROS detoxification, and osmotic adjustment through the synthesis of organic osmolytes (Azooz, 2009; Munns and Tester, 2008; Chen et al., 2018; Yancey, 2005; Misra and Saxena, 2009).

Salicylic acid (SA), a phenolic plant growth regulator, plays a crucial role in regulating various physiological processes and enhancing plant defense against abiotic stresses (Tahjib-Ul-Arif et al., 2018). Considerable research has highlighted the protective role of SA against multiple stress conditions, primarily through modulation of antioxidant systems and regulation of ROS levels (Sakhabutdinova et al., 2003; Shakirova et al., 2003; Mahmud et al., 2017). The effects of SA on cellular and molecular metabolism vary depending on plant species, SA concentration, environmental conditions, and method of application (El-Mergawi and Abd El-Wahed, 2020).

SA also acts as an important signaling molecule in plant stress responses, particularly under salinity conditions (Jayakannan et al., 2015). It enhances plant tolerance to salt stress by improving resistance to ion toxicity and oxidative damage, stimulating the ascorbate–glutathione (AsA–GSH) antioxidant pathway, and preventing severe reductions in plant growth (Hasanuzzaman et al., 2019). Numerous studies have demonstrated that exogenous application of SA alleviates salt stress in many fruit and vegetable crops, although its effectiveness may vary depending on plant species, growth conditions, and application strategies. Furthermore, interactions between salicylic acid (SA) and jasmonic acid (JA) signaling pathways contribute to improved salt tolerance by regulating Na^+^ and K^+^ balance in plant tissues. These hormonal interactions also play an important role in enhancing crop productivity, highlighting their potential as valuable tools for sustainable agriculture (Mana et al., 2023; Asensi-Fabado and Munné-Bosch, 2011).

## 3 Jasmonic acid and tolerance of salinity stress in plants

Jasmonic acid (JA) was initially identified as a stress-associated phytohormone in higher plants; however, it is now recognized as a pivotal endogenous signaling molecule that regulates diverse aspects of plant growth, development, and environmental adaptation. Since its discovery in 1962, extensive research has established its central role in coordinating key biological processes, including cellular differentiation, defense signaling, and reproductive development (Shekhawat et al., 2023). Chemically, jasmonates are lipid-derived compounds belonging to the oxylipin family, characterized by cyclopentanone structures formed via the octadecanoid pathway. The term “jasmonates” broadly encompasses JA and its bioactive derivatives.

Jasmonates comprise a structurally diverse group of signaling molecules derived from jasmonic acid, including methyl jasmonate (MeJA), cis-jasmone, jasmonoyl-isoleucine (JA-Ile), 12-hydroxy-JA-Ile lactones, and glucosylated conjugates such as 12-O-glucosyl-JA-Ile (Kohli et al., 2019). Among these, JA-Ile is considered the primary bioactive ligand involved in receptor-mediated signaling. The endogenous concentration of JA varies spatially and temporally across plant tissues, typically remaining low in vegetative organs such as mature leaves and roots, while accumulating to higher levels in reproductive tissues, particularly flowers (Kanwar et al., 2020). Functionally, JA regulates multiple physiological and biochemical processes, including modulation of photosynthetic electron transport, nutrient uptake (notably nitrogen and phosphorus), stomatal dynamics, carbohydrate partitioning, and cell cycle progression (Pandey, 2018). In addition, JA plays a critical role in fruit development and ripening. Its volatile derivative, MeJA, has been widely studied for its capacity to enhance fruit quality attributes and stress tolerance. In climacteric fruits such as apple, exogenous MeJA application promotes anthocyanin and carotenoid biosynthesis, enhances phenolic accumulation, and stimulates ethylene production, thereby improving coloration, aroma, and ripening processes (Ni et al., 2020). Conversely, in non-climacteric fruits such as strawberry, raspberry, and blackberry, MeJA induces distinct physiological responses, including increased anthocyanin accumulation, improved soluble solids content to titratable acidity (SSC/TA) ratio, and enhanced sugar metabolism (Wang et al., 2008). At the molecular level, JA signaling is tightly regulated through transcriptional networks. The basic helix–loop–helix transcription factor MYC2, encoded by the JIN1 gene, acts as a central regulator of JA-responsive gene expression by binding to G-box (5′-CACGTG-3′) elements in promoter regions (Poonam et al., 2013). This interaction activates downstream defense-related genes involved in stress adaptation. Jasmonates are known to enhance plant tolerance to abiotic stresses primarily through the induction of antioxidant defense systems, accumulation of osmoprotectants, and activation of stress-responsive signaling pathways. However, under severe stress conditions, the expression of JA-responsive genes may be suppressed, thereby limiting the efficiency of JA-mediated defense responses, as observed in tomato under high-stress environments (Bali et al., 2018; Allagulova et al., 2020). Exogenous application of jasmonates has been demonstrated to mitigate the detrimental effects of abiotic stresses in various crop species. For instance, foliar application of JA can enhance antioxidant enzyme activities, improve photosynthetic efficiency, and regulate ion homeostasis, thereby alleviating stress-induced damage (Zeeshan et al., 2023). Nonetheless, JA-mediated responses are often associated with growth inhibition, particularly during early developmental stages, where reduced cell elongation and increased fruit abscission have been reported (Avalbaev et al., 2021). Under stress conditions, such as in Brassica parachinensis, significant reductions in biomass accumulation, root and shoot elongation, and increased cellular damage have been observed. However, JA treatment can counteract these adverse effects by enhancing detoxification mechanisms, reducing heavy metal accumulation (e.g., chromium), improving gas exchange parameters, and stabilizing photosynthetic pigments (Lymperopoulos et al., 2018).

Salinity represents one of the most critical abiotic stresses limiting agricultural productivity worldwide, affecting approximately 10% of global arable land (Zhu et al., 2024). High salinity disrupts plant water relations by lowering soil water potential, induces ionic imbalance, and interferes with essential physiological processes, including nutrient uptake, respiration, transpiration, and chlorophyll biosynthesis (Ulloa-Inostroza et al., 2017). Moreover, salinity stress leads to excessive generation of reactive oxygen species (ROS), resulting in oxidative damage to proteins, lipids, nucleic acids, and enzymatic systems, ultimately causing membrane destabilization and increased lipid peroxidation (Bali et al., 2019). Jasmonates play a crucial role in mediating plant responses to salinity stress by modulating physiological, biochemical, and molecular processes. Endogenous JA levels have been shown to increase under saline conditions, as reported in Iris hexagona, suggesting its involvement in stress perception and signaling. Exogenous JA application enhances plant tolerance to salinity by improving biomass accumulation, maintaining ion homeostasis (particularly K^+^/Na^+^ balance), and stimulating antioxidant defense systems (Wasternack and Strnad, 2019). In crops such as rice and soybean, JA treatment has been associated with improved growth performance, increased chlorophyll content, enhanced relative water content, and higher yield under saline conditions. Additionally, JA has been shown to regulate lipid metabolism by increasing linoleic and linolenic acid content through the upregulation of lipoxygenase activity, thereby improving membrane stability and oil quality (Shirani Bidabadi and Sharifi, 2021). Furthermore, JA influences ion transport mechanisms, including the activation of H^+^-ATPase activity in root systems, which contributes to reduced sodium uptake and improved ionic balance under salt stress (Ndiaye et al., 2022). Foliar application of JA has also been reported to enhance potassium retention, delay K^+^ depletion, and reduce lipid peroxidation, thereby strengthening plant resilience under saline environments. Despite significant advancements, the molecular mechanisms underlying JA-mediated stress responses remain incompletely understood. Future research should focus on elucidating JA-specific signaling pathways, identifying key target genes, and uncovering the crosstalk between jasmonates and other phytohormonal networks. Such insights will be essential for optimizing the use of JA in improving crop tolerance to abiotic stresses, particularly salinity, under changing climatic conditions.

A comprehensive understanding of jasmonate signaling necessitates consideration of the COI1–JAZ signaling module, which represents the canonical mechanism underlying jasmonic acid (JA) perception and signal transduction in plants. In this pathway, the bioactive conjugate jasmonoyl-isoleucine (JA-Ile) functions as a ligand that promotes the interaction between the F-box protein CORONATINE INSENSITIVE 1 (COI1) and JASMONATE ZIM-DOMAIN (JAZ) repressor proteins. COI1 operates as a key component of the SCF^COI1 (SKP1–Cullin–F-box) E3 ubiquitin ligase complex, facilitating ubiquitin-mediated proteasomal degradation of target proteins (Wasternack and Strnad, 2019). Under basal conditions, JAZ proteins repress JA-responsive gene expression by physically interacting with transcription factors such as MYC2, while simultaneously recruiting co-repressors like TOPLESS (TPL) through adaptor proteins including NINJA, thereby forming a tightly regulated transcriptional repression complex. Upon stress-induced accumulation of JA-Ile, the ligand acts as a molecular glue that stabilizes the COI1–JA-Ile–JAZ co-receptor complex, triggering ubiquitination and subsequent degradation of JAZ repressors via the 26S proteasome pathway.

**Figure 1:**
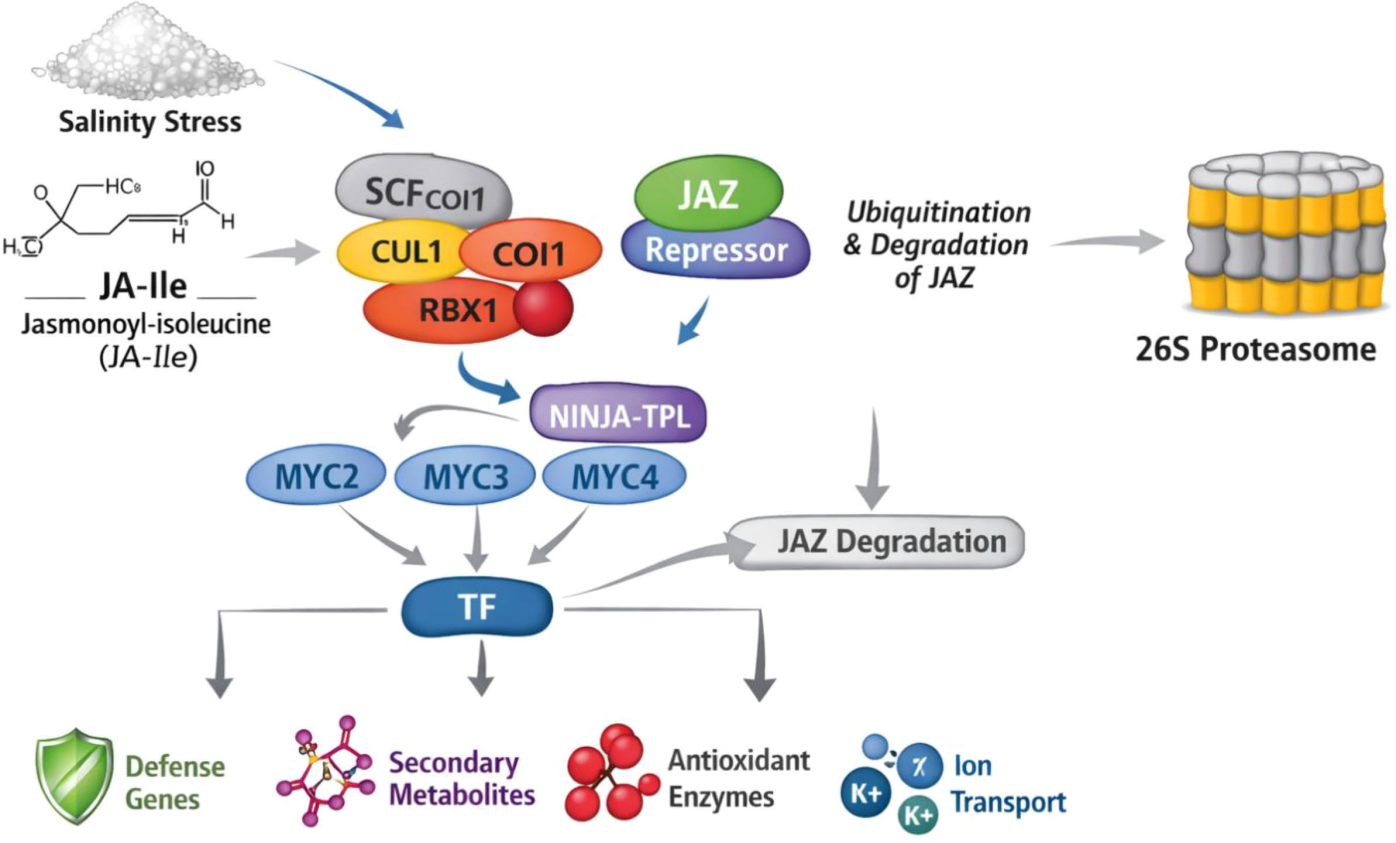
Conceptual model of JA signaling under salinity stress.

The removal of JAZ-mediated repression liberates transcription factors such as MYC2, enabling their binding to G-box (5′-CACGTG-3′) elements in the promoters of target genes and initiating transcriptional activation of JA-responsive pathways (Poonam et al., 2013). These downstream responses encompass the regulation of antioxidant defense systems, secondary metabolite biosynthesis, and stress-responsive gene networks. Notably, the COI1–JAZ module is subject to negative feedback regulation, as JAZ genes themselves are induced by JA signaling, ensuring rapid attenuation and homeostatic balance (Chini et al., 2007; Çelikten et al., 2019). Furthermore, this signaling cascade is intricately integrated with other phytohormonal pathways, including abscisic acid, ethylene, and salicylic acid, allowing coordinated regulation of plant growth, development, and stress adaptation (Pieterse et al., 2012; Wasternack and Strnad, 2019). Collectively, the COI1–JAZ signaling module functions as a highly conserved molecular switch that enables plants to perceive jasmonate signals and rapidly reprogram gene expression in response to environmental stimuli (**Figure 1**).

## 4 Salicylic acid and tolerance of salinity stress in plants

SA is a crucial component of plants’ complex signaling system. SA, a naturally occurring phenolic molecule, is an essential hormone present in plants that regulates several physiological processes necessary for environmental adaptation, plant growth, and development. It is derived from the phenylpropanoid pathway and has a low molecular weight (Rai et al., 2024). Different plant species contain this molecule in different amounts. In SA biosynthesis, a series of enzyme events convert chorismic acid, a precursor in the shikimate pathway, into SA. Chloroplasts and mitochondria are where these processes mostly occur. Abiotic stressors frequently increase the production of SA in particular as a plant defense strategy (Lefevere et al., 2020). SA is not dispersed equally throughout the plant; instead, it builds up in particular cell compartments and tissues. Its distribution is influenced by environmental factors and developmental phases. It is essential to comprehend the temporal and spatial distribution of SA in order to explain its diverse functions in defense and stress response (Nanda et al., 2022). Complicated signaling mechanisms mediate SA’s biological activities.

**Figure 2:**
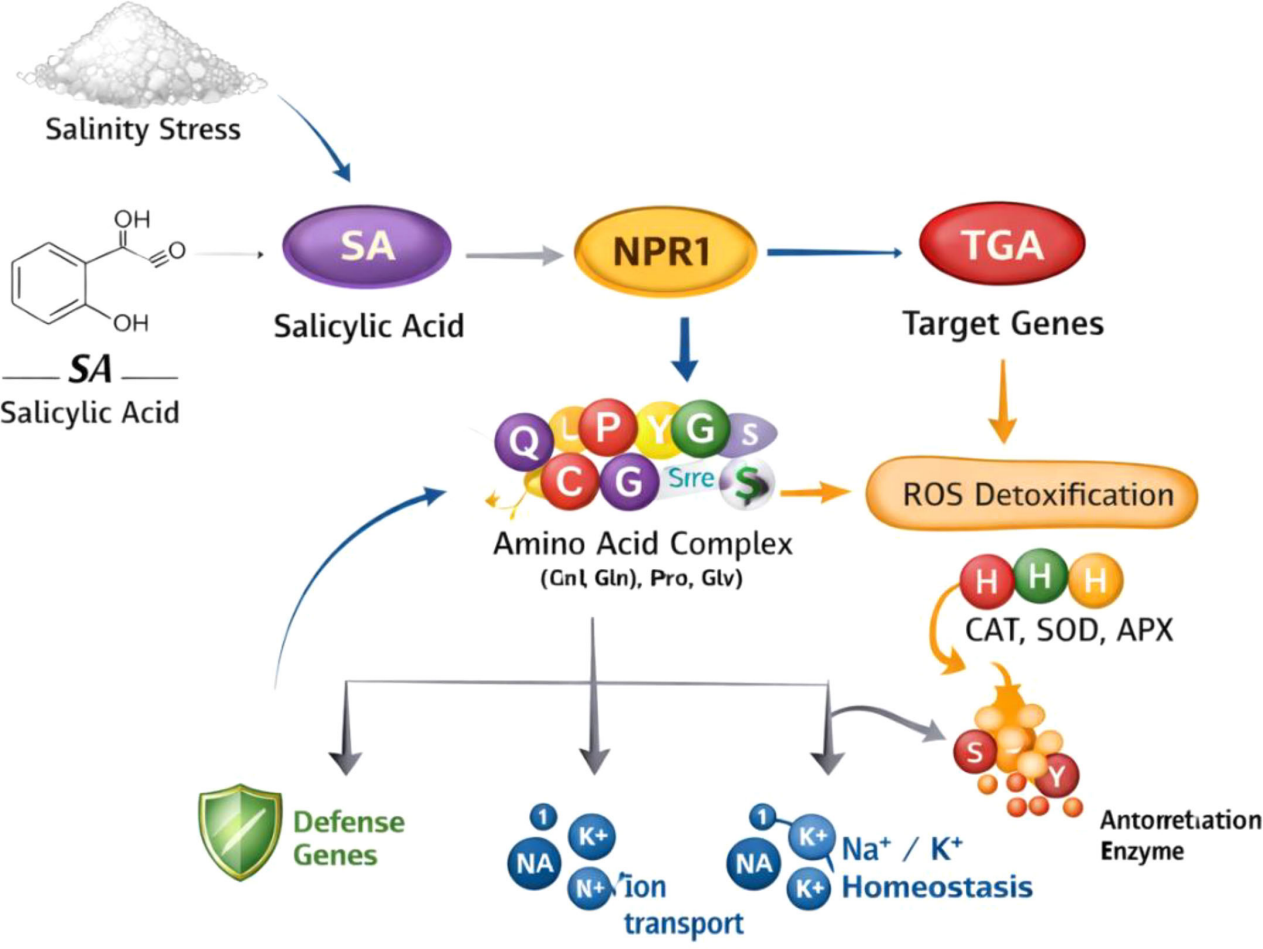
Conceptual model of SA signaling under salinity stress.

Actually, given that salt stress throws off cellular ionic and osmotic balances, it lowers plant productivity (Alvan et al., 2025). The main negative effects of salinity stress are decreased stomatal conductance, osmotic stress, nutrient uptake and homeostasis/deficiency, and increased oxidative stress from ROS. Furthermore, the leaves’ water potential decreased, their cell turgor decreased more, and biochemical and physiological processes changed (Faizan et al., 2024; Karabulut, 2024). Many crops, including Brassica juncea (Nazar et al., 2015), Medicago sativa, *Vicia faba* (Azooz, 2009), and *V. radiata*, have shown a strong correlation between SA and improving mechanisms for coping with salinity stress. SA’s higher chlorophyll content and antioxidant enzyme activity have been demonstrated to boost biomass and enhance Torreya grandis’s tolerance to salinity. Thus, oxidative stress is decreased and photosynthesis is stimulated (Li et al., 2014). In SA-deficient NahG transgenic Arabidopsis lines, the main consequences of SA deficiency in plants have been found to be decreased antioxidant enzyme activity and increased salinity-induced damage (Li et al., 2014; Cao et al., 2009). By reducing cellular malondialdehyde and ROS, SA (50 μM) reduced the amount of oxidative stress that was accumulated in *Hordeum vulgare*. To increase the activity of key enzymes that metabolise H_2_O_2_ based on GSH, like GST, SA-priming might be a useful tactic. The SA enhanced the ability of *Arabidopsis thaliana* to withstand salinity by re-establishing membrane potential and blocking the destruction of K^+^ by salt through a GORK channel (Jayakannan et al., 2015). Most often, a plant’s normal aerobic metabolism produces and scavenges a variety of ROS. Specifically, low amounts of ROS may be able to activate or regulate plant responses to different stressors and play significant signaling roles. But oxidative stress, a physiological state, is the end result of (abiotic) stressors that cause the production and scavenging of ROS to be out of balance. Uncontrolled oxidative stress can therefore have a variety of negative effects, including cell death, oxidative alteration of essential macromolecules, and halting plant development and growth (Faizan et al., 2024). Apoplastic ROS have been shown to control cell death by interacting with various signaling pathways, including those that are mediated by SA (Overmyer et al., 2003). It has been demonstrated that both exogenous and endogenous SA tightly control cellular ROS and contribute to antioxidant metabolism. However, an appropriate defensive response was obtained by combining ROS signaling with both independent and SA-dependent signaling components (**Figure 2**). Additionally, it causes a protein kinase to become activated (Spoel and Dong, 2024). The plant-abiotic stress tolerance system and SA may offer acquired resistance (Song et al., 2023). There was proof that SA is a crucial signaling molecule needed for *A. thaliana*’s GSH-based defense gene expression to be triggered by the agonising zone (Rao and Davis, 1999). Because ROS and SA signaling have an antagonistic effect on apoplastic ROS signaling*, A. thaliana* plants can detect the response of the ROS-SA interaction. Along with its well-known role in regulating defensive reactions, SA signaling has also been shown to control light adaption mechanisms, which in turn affect photosynthetic function (Gawroński et al., 2013). SA controls the antioxidants’ metabolism, which in turn controls plant resistance to significant abiotic stressors such as heat, metal, UV-B rays, osmotic stress, and ozone. SA-priming influenced the GST-supergene family in S. lycopersicum in a concentration-dependent way. It was suggested that Plants’ resistance to inozone would be increased by SA-mediated activation of SOD and GSH-based H_2_O_2_-metabolizing enzymes, such as GPX and GST (Milla et al., 2003). In a different instance, applying SA-induced SOD activity caused a brief increase in H_2_O_2_ levels and a rise in shoot Ca^2+^, a second messenger, which was believed to activate antioxidant enzymes and ultimately reduce ROS in cells (Arfan, 2009).

Remarkably, a transcriptome profiling investigation that contrasted a salt-sensitive tomato cultivar with wild halophytic tomatoes found that only in wild tomatoes was SABP2 activated by salinity, suggesting that SABP2 is connected to salt tolerance pathways (Sun et al., 2010). Nevertheless, it is unknown exactly which SABP2-mediated signaling occurs during salt stress (Attaran and He, 2012).

**Figure 3:**
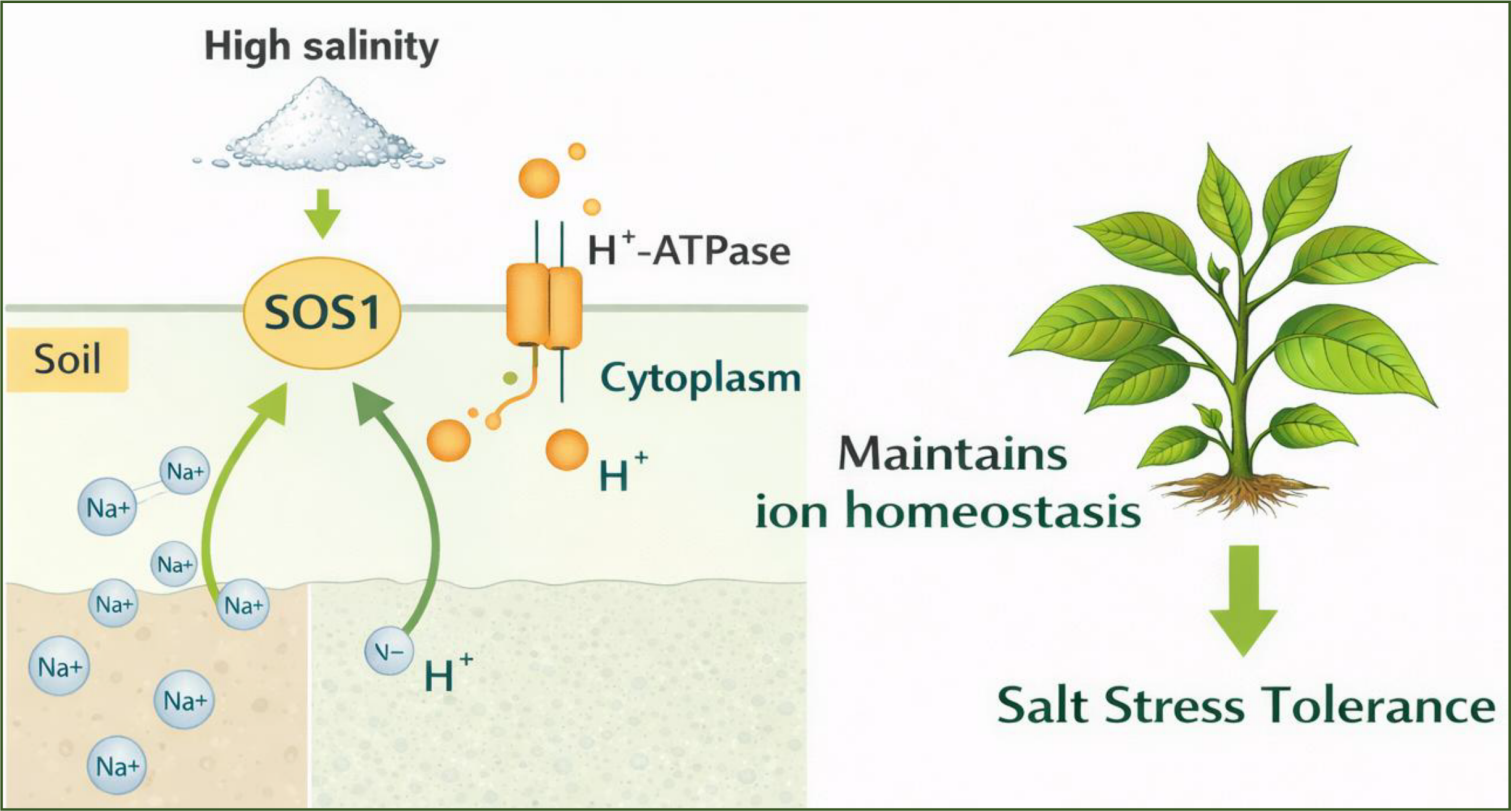
The role and importance of Na^+^ transport in SOS1 on plants.

Redox equilibrium is maintained in plants by the proper ratio of ROS generation to scavenging. Low amounts of SA generally help people tolerate abiotic stressors. However, high levels increase the production of ROS species, which kill cells and cause oxidative stress (Faizan et al., 2024). High concentrations of H_2_O_2_, a ROS species, cause cell death that resembles autophagy and apoptosis, but at low concentrations, they function similarly to SA as a signaling molecule to produce tolerance to a range of abiotic stimuli (Faizan et al., 2024). Several transporters aid in the absorption of Na^+^ under salt stress. Both high-affinity Na^+^ absorption and internal Na^+^ redistribution are facilitated by the high-affinity potassium transporters (HKT) present in many plant species (Munns et al., 2012). SA pretreatment and the high-endogenous-SA mutant nudt7 dramatically decreased the concentration of Na+ in Arabidopsis wild-type shoots after extended salt stress (Jayakannan et al., 2013). In comparison to the wild type, another high-affinity mutant gh SA (npr1-5) stored more Na+ and showed hypersensitivity to salt stress. This is essential for tolerance to salt. The decreased Na^+^ in shoots is still unknown, though, and may be caused by either enhancing Na^+^ removal from the xylem or inhibiting Na^+^ loading of the shoots. Na^+^ entry in Arabidopsis roots under acute salt stress was not significantly altered by a 1-hour exogenous SA pretreatment (Jayakannan et al., 2013). According to the aforementioned findings, exogenous SA acts on Na^+^ transporters at the post-transcriptional level and takes more than an hour to have an effect. Despite measuring net Na^+^ fluxes in the aforementioned study, determining whether SA enhances the activity of Na^+^/H^+^ exchangers or inhibits Na+ entry pathways is difficult. To overcome this issue, more experiments are required. Strong voltage-dependent non-selective cation channels (NSCC) are assumed to be the main mechanism by which Na^+^ enters roots exposed to high concentrations of NaCl (Horie et al., 2006). Two subgroups of NSCC channels can mediate Na^+^ uptake in plants: Cyclic nucleotide-gated channels and glutamate receptor-like channels (GLRs). It may be suggested that the latter are potential downstream targets of SA. In fact, tomato leaves’ glutamate synthase activity was increased by salt stress, while exogenous SA controlled the activity of glutamate dehydrogenase in maize roots. Thus, it makes sense to hypothesise that SA might control GLRs that are involved in plants’ entrance and redistribution of Na^+^ (Zhu, 2003). Nearly every tissue has been shown to have SOS1 promoter activity. However, the cells that are most active are the root epidermal cells, especially those that surround the vascular tissue and the tips of the roots. Among other things, the transport of sodium ions from the cytosol to the rhizosphere depends on SOS1 (**Figure 3**). Delaying the cytoplasmic Na^+^ accumulation allows for more time to be spent storing Na^+^ in the vacuole. Additionally, it controls the long-distance flow of Na^+^ through Na^+^ recovery between roots and shoots (Zhu, 2003). An essential part of the mechanism used to tolerate salinity by salt-tolerant species, such as halophytes, vacuolar Na^+^ sequestration is necessary to maintain low cytosolic Na^+^ concentrations during sodium transport over the tonoplast (Shabala, 2013).

The CPA family of cation/proton antiporters includes tonoplast Na^+^/H^+^ exchangers (NHX) (Apse et al., 1999), which mediate the sequestration. Arabidopsis contains at least six different types of NHX isolates, each with unique expression patterns that vary according on the tissue and the stress. These findings demonstrate that vacuoles’ enhanced ability to sequester Na^+^ is crucial for their ability to withstand salt. Interestingly, Na^+^/H^+^ exchange and K^+^ transport can be mediated by AtNHX2 and AtNHX1 (Barragán et al., 2012). While there isn’t any concrete proof that SA controls NHXs, ABA and/or SA treatments increase the expression in a number of plant species (Guan et al., 2011). In addition to preventing K^+^ loss from NaCl, it enhanced salt tolerance. SA decreased the negative effects of salt (Hayat et al., 2010) and raised the K^+^ content in the roots of a number of plant species (He and Zhu, 2008). However, it was unclear if the prevention of K^+^ loss or increased K^+^ intake was primarily responsible for this beneficial effect (Jayakannan et al., 2013). Interestingly, the amount of K^+^ that leaked through the GORK channel was reduced when Arabidopsis roots were pretreated with a physiologically realistic concentration of SA (<0.5 mM). GORK is an essential voltage-gated channel for regulating membrane potential, and it is significantly impacted by plasma membrane H^+^-ATPase (Palmgren and Nissen, 2010). In species that can withstand salt, the effect is more pronounced than in species that cannot (Jayakannan et al., 2013). The activation of proton pumps triggered by salt stress was positively correlated with salinity tolerance (Kerkeb et al., 2001). A rise in H^+^ pumping of this type could have two concurrent effects. First, depolarization-activated outwardly rectifying K^+^ channels would be down-regulated by increased H^+^-ATPase activity, blocking K^+^ leakage through KOR channels (Jayakannan et al., 2013). H^+^ pumping lowers the cytoplasm’s Na^+^/K^+^ ratio, which triggers the plasma membrane Na^+^/H^+^ exchanger (SOS1) to transfer Na^+^ from the cytoplasm to the apoplast (Apse and Blumwald, 2007). In grape and pea under heat stress, pretreatment with SA increased plasma membrane H^+^-ATPase activity (Liu et al., 2009); therefore, SA may have an impact on both of the aforementioned mechanisms. All things considered, increased plasma membrane H^+^-ATPase activity and its consequent effects on intracellular K^+^ and Na^+^ ionic equilibrium seem to be linked to SA’s advantageous effects under salt stress. K^+^ permeable channels induced by ROS are another significant mechanism for K^+^ leakage from the cytosol in saline environments. Cellular compartments that produce various types of ROS under salt stress include the apoplast, mitochondria, and chloroplasts (found in leaves) (Miller et al., 2009). When under salt stress, certain ROS species (OH and H_2_O_2_) can activate GORK or NSCC channels (Demidchik et al., 2010), which results in K^+^ depletion and programmed cell death. In order to prevent K^+^ loss through NSCC, which is triggered by ROS, plants must be able to withstand salt stress. Through ROS-activated NSCC, SA can control K^+^ loss due to the known interaction between SA and ROS signaling pathways. The two Arabidopsis mutants (nudt7 and npr1-5) with altered SA signaling and elevated endogenous SA concentrations were the only ones in which SA decreased oxidative stress and oxidative damage. This conclusion was proposed because the npr1–5 mutant was more sensitive to ROS stress and exhibited higher K^+^ efflux than the nudt7 mutant (Jayakannan et al., 2013).

Through the control of other plant hormone production, SA can control plant resistance (Chen et al., 2021). Inhibiting JA methylation and ETH production, for instance, has been shown to decrease a plant’s ability to withstand adversity. By interfering with other plant hormones’ signaling pathways, SA can either make a plant more or less resilient to stress (Gutierrez-Larruscain et al., 2022). For instance, by triggering or blocking hormone signaling pathways, SA can control plant tolerance. A well-known signaling molecule, SA is believed to be an essential stress hormone for plants. It is essential for both robust plant growth and susceptibility to stress. Plants’ regular growth and defense against unfavourable environmental conditions are largely dependent on SA’s function in abiotic stress tolerance, as demonstrated by numerous studies. A variety of physiological and morphological responses are initiated by SA, a signaling molecule that enables horticulture plants to tolerate abiotic stress (Yang et al., 2023). SA may compete with other plant hormones to either activate or inhibit shared signaling pathways or components. Thus, it has the ability to control the plant’s ability to tolerate stress. This competition may lead to dominant or complementary effects of hormone signaling that affect the plant’s response to adversity. SA is a vital signaling molecule that initiates reactions to abiotic stress and takes part in developmental signaling pathways that govern and control a variety of physiological and biochemical processes in normal plant growth, development, maturation, flowering, and senescence (Sedláková et al., 2023). Plants are protected from heat stress by NO and SA through increasing sulfur uptake, decreasing oxidative stress, and increasing antioxidant enzyme activity (Afzal et al., 2020). SA content alone cannot account for the positive effects of willow extract treatments, even though SA can improve salinity tolerance. Pure SA applied exogenously did not yield the same effects (Mutlu-Durak et al., 2023). At specific dosages, SA-Ricemate can be used as a foliar spray to 71% lessen the severity of bacterial leaf blight disease. In the field, SA-Ricemate enhanced total grain yield and dramatically decreased disease severity by 78% (Thepbandit et al., 2023). In plants treated with Ni, SA and SNP supplementation enhanced root and shoot length, dry mass, chlorophyll content, and mineral concentration while notably mitigating the negative effects of Ni. Millet plants are protected against oxidative damage brought on by Ni application by exogenous SNP or SA application, especially SNP and SA combined (Kotapati et al., 2017). Salt toxicity may be lessened in sustainable agricultural systems more effectively by co-applying SA and spermine (SPM). While reducing the negative effects of salt stress, exogenously applied SA and/or SPM significantly increased wheat growth and yield by enhancing photosynthetic pigment content, osmolyte accumulation, ionic homeostasis, nutrient acquisition, and protein content (Talaat and Hanafy, 2023). Co-applying SA with essential plant nutrients is a realistic, affordable, and accessible method of reducing the adverse effects of deficiency irrigation stress. Additionally, under typical irrigation conditions, this method demonstrated further gains in wheat productivity and growth (Alotaibi et al., 2023). Numerous physiological functions in plants, including disease resistance and phytohormone signal transduction, depend on MYB transcription factors (Zhang et al., 2022). N-methyltransferase (NMT) genes have been demonstrated to be less repressed by exogenous applications of SA and MeJA. Plant growth, development, and stress responses are all regulated by these genes, which are crucial for plant survival and adaptation (Kumar and Giridhar, 2015). This suggests that signaling cascades that result in overexpression of the NMT gene in mature endosperm due to SA and MeJA treatment and those that regulate its expression during maturation may interact (Kumar and Giridhar, 2015). Finally, through mechanisms like hormone synthesis control, signaling pathways, gene expression, and hormone competition, SA’s interaction with other plant hormones either increases or decreases plant tolerance. The type of stress, the plant species, and the hormone interaction setting will all affect the specifics of these systems (Ameen et al., 2025).

A major global issue, salinity stress severely limits plant growth, lowers production, and negatively impacts seedling vigour and root development. Plant metabolism is altered by this stressor, which results in nutrient shortages, membrane abnormalities, and genetic damage (Atta et al., 2023). Furthermore, oxidative stress can result from salt stress’s increased generation of reactive oxygen species, including singlet oxygen, hydrogen peroxide (H_2_O_2_), and free radicals. One study discovered that SA decreased plant uptake of sodium while increasing the absorption of essential nutrients such as magnesium (Mg), phosphorus (P), potassium (K), calcium (Ca), and nitrogen (N) when compared to control treatments during salt stress (Meena et al., 2020). Therefore, SA can improve a plant’s resistance to salt stress both naturally and through external application. It was noted in an Egyptian study that throughout the summer, sunflower plants were cultivated in saline soil (El Sabagh et al., 2022). JA and SA have a complex relationship. Although they are typically adversarial, there are times when they can work together. JA is more closely linked to defense mechanisms against necrotrophic pathogens and herbivores, whereas SA is more closely linked to biotrophic pathogens. For the plant to respond to particular stressors, the balance between these two hormones is crucial (Roychowdhury et al., 2024). Through intricate signaling channels and cross-talk mechanisms, these connections allow plants to adjust how they react to various stresses (**Table 1**).

**Table 1:** The responses of JA and SA roles on plants in sality stress.

Plant species	Condition and dose of stress	Results and their implications for antioxidant systems	References
Wheat (*Triticum aestivum* L.)	150 mM NaCl; 3 days	An external source of JA increased stress tolerance by lowering MDA and H_2_O_2_ levels, increasing SOD, POD, CAT, and APX activities, and increasing GSH, Chl b, and carotenoid concentrations.	Qiu et al., 2014.
Mustard (*Brassica carinata* L.)	NaCl 50, 100, and 150 mM; 9 weeks	Foliar SA supplementation enhanced SOD (29–32%), CAT (27–25%), and POX (179–194%) efficiency while mitigating the salinity effect.	Husen et al., 2018.
Rice (*Oryza sativa* L.)	NaCl at 0, 100, 200, 300 and 400 mM L^−1^	Through the modulation of antioxidant system activities, SA mitigated the negative effects of salinity; SOD, CAT, and POX increased under salt stress and decreased when SA was applied.	Jini and Joseph, 2017.
Pomegranate (*Punica granatum* L.)	10, 35, and 70 mM NaCl	SA treatment reduced MDA content, electrolyte leakage (EL), Na, and Cl levels while increasing total phenolic, chlorophyll, carbohydrate, and proline contents as well as POD and CAT activities.	Khalil et al., 2022.
Soapwort (*Saponaria officinalis* L.)	100, 200, and 300 mmol L^−1^ NaCl solution	SA improved salt tolerance by modifying ion homeostasis, osmoprotectants, photosynthetic rate, and antioxidant levels.	Xu et al., 2022.
Barley (*Hordeum vulgare* L.)	0.5 mM	The effectiveness of SOD, CAT, POX, and K^+^/Na^+^ was improved by JA treatment, which raised stress tolerance.	Pakar et al., 2016.
Zingiber officinale Roscoe	Concentrations of NaCl (0, 50, 100, 150 and 200 mM). Observations recorded after 14 days.	SA’s role as a signaling molecule has been connected to the activation of the plant’s defense mechanisms, including osmoregulation, ROS scavenging, ion homeostasis, etc.	Hundare et al., 2022.
*Zea mays* L.	4 ve 8 dS m^−1^, NaCl	In the presence of salt stress, SA produced greater biomass output and improved growth when applied exogenously.	
*Zea mays* cv. Caramelo F1	60 mM NaCl, 30 DAS	Applications of SA have been shown to dramatically lower the concentration of Na in maize under salinity stress.	Mutlu-Durak et al., 2023.

Depending on the specific plant species involved and the type of stress, the results of these interactions can differ. For instance, during a biotrophic pathogen attack, SA frequently builds up to trigger defensive mechanisms, but ethylene and JA may be suppressed to put resistance ahead of growth. Nonetheless, the balance between ABA and SA is crucial for regulating stomatal closure, preserving water, and minimising damage during abiotic stressors (Rai et al., 2024).

## 5 Mechanisms of salicylic acid and jasmonic acid-mediated stress mitigation

To fully utilise SA’s potential to increase crop resilience, it is essential to comprehend how it influences stress tolerance systems. By altering gene expression, SA mostly improves resistance to abiotic stress (Elsisi et al., 2024). The production of stress-related proteins, enzymes, and osmoprotectants is impacted by SA-induced alterations in gene expression patterns. By preserving cellular homeostasis and encouraging stress adaption, this in turn aids plants in managing stress. Oxidative damage is often caused by the overproduction of ROS by plant cells under abiotic stress. The synthesis of SOD, CAT, and POD, among other antioxidant enzymes, is promoted by SA. These enzymes reduce oxidative stress and preserve cell integrity by neutralising ROS. Osmotic imbalances and water shortages are common in plants under abiotic stress (Singh and Satheeshkumar, 2024). Salicylic acid regulates the synthesis of key osmoprotectants, including proline, soluble carbohydrates, and polyamines, which play a crucial role in maintaining osmotic balance under stress conditions. These osmolytes contribute to the preservation of cellular hydration, stabilization of macromolecules, and maintenance of turgor pressure. Recent studies provide a comprehensive overview of the interactions between SA and other phytohormones, major osmolytes, mineral nutrients, and secondary metabolites, as well as its role in reactive oxygen species (ROS) signaling and antioxidant regulation, thereby elucidating the mechanisms underlying SA-mediated enhancement of abiotic stress tolerance in plants. The interaction of SA with osmolytes significantly contributes to the activation of plant defense systems against both osmotic and oxidative stress. Osmolyte-mediated osmotic adjustment represents a highly evolved adaptive mechanism that enables plants to maintain cellular homeostasis and functional integrity under adverse environmental conditions (Elsisi et al., 2024). These substances help stressed plants maintain their turgor and avoid interfering with the metabolic processes of other plants (Misra and Saxena, 2009). It is believed that glycinebetaine is a great solute for osmotic adjustment and defense against osmotic stress (Munns, 2005), heat stress (Wang et al., 2010), metal stress (Bharwana et al., 2014), and salt stress (Khan et al., 2012). According to Ashraf and Foolad (2007), plants that accumulate under GB stress retain Rubisco activity, stabilise membrane integrity, prevent polypeptides from dissociating from the PSII complex, regulate cell osmotic balance, and detoxify harmful chemicals. Specifically, plants under high NaCl, drought, and cold stress conditions can accumulate GB within 0.5–2.5 mM in when exposed to SA and its counterpart aspirin (Jagendorf and Takabe, 2001). Both aspirin and salicylate are effective at causing GB buildup, which is a component of systematically acquired resistance. When plants experience drought stress, NaCl might play a significant role. Another effect of GB induction could be the activation of protein kinase in response to hyperosmotic stress (Hoyos and Zhang, 2000). Overall plant development can be enhanced by an SA-mediated rise in GB level (Misra and Misra, 2012). The increase in GB content led to an increase in *Rauwolfias serpentine* biomass. GB contributes to the decrease in photosynthesis and growth inhibition brought on by SA in *V. radiata*. Under salt stress, SA (at 0.5 mM) was demonstrated to prevent the generation of excess ethylene and cause GB buildup through an increase in methionine concentration. The detrimental impacts of salinity on photosynthesis and growth were mitigated, according to these authors, when the SA analogue 2, 6, dichloro-isonicotinic acid was applied to GB accumulation. Pro, another crucial rosmolyte, is accumulated by plants as one of their adaptive strategies for survival, particularly in situations of osmotic stress and salt. It preserves biological membranes, stabilises enzymes and proteins, controls cellular osmotic balance, and detoxifies excess ROS (Iqbal et al., 2014).

Research has shown that SA can increase prometabolism in the face of abiotic stressors (Misra and Saxena, 2009; Khan et al., 2013). During salinity stress, SA (at 0.5 mM) significantly increased the amount of Pro and activated enzymes involved in Probiosynthesis, including pyrroline-5-carboxylate reducase and γ-glutamylkinase. *Lens esculenta* ability to withstand salt stress was credited with this rise in prometabolism (Misra and Saxena, 2009). The down-regulation of Prooxidase activity and the up-regulation of Probiosynthesis enzymes (such as pyrroline-5-carboxylatereductase and γ-glutamylkinase) were shown to be the causes of elevated Pro levels in SA and plant abiotic stress tolerance. On the contrary, increased Pro levels were consistent with the maintenance of R. serpentina cell turgor under salt stress (Khan et al., 2013). Increased Pro production was suggested as a way to improve nitrogen uptake and lessen the negative effects of heat stress on photosynthesis. However, exogenous Pro application has been demonstrated to alter the calcium (Ca)-mediated oxidative burst defense response in plants and trigger SA production (through the signaling pathway that is dependent on NDR1) (Chen et al., 2011). Additionally, soluble sugar and the buildup of sugar alcohol-mannitol have been shown to act as osmotic defenders, helping plants to withstand stress (Cheng et al., 2009).

SA mineral nutrition is crucial for both healthy growth and development as well as survival in a variety of harsh environmental conditions. According to research, plants’ mineral nutritional status can be sacrificed to reduce abiotic stress (Yadav et al., 2024). The metabolism and absorption of vital mineral elements can be considerably regulated by SA. Thus, it can help abiotically stressed plants grow and develop (Yadav et al., 2024). According to a different study, SA (0.5 mM) mediates the maintenance of high K^+^/Na^+^ and Ca^2+^/Na^+^ ratios, which are necessary to improve *Z. mays* growth, gas exchange, yield, and salinity tolerance (Tufail et al., 2013). Nazar et al. (2015) showed that by increasing S assimilation, SA can increase B. juncea’s tolerance to salinity. Calcium is a crucial element that is known to play a number of structural roles in plants under both favourable and stressful conditions. There is proof of a strong correlation between SA and Ca (and Ca signaling) in stressed plants (Faizan et al., 2024b). The connection between SA and Ca^2+^ signaling may affect the preservation of K^+^/Na-ion selectivity and stress-induced defense mechanisms (Chen et al., 2001). Pro levels can be raised by SA and Ca alone or in combination, which greatly increases plants’ resistance to salinity (Al-Whaibi et al., 2012). Pro has also been connected to SA signaling and the Ca-mediated oxidative burst production process (Chen et al., 2011). Abiotic stress responses have been demonstrated to involve Ca-dependent protein kinases (CDPKs), which SA can also trigger. SA inhibited K^+^ channel activity, which resulted in stomatal closure, however it was unable to cause Ca-Cytosylations in guard cells (Yu et al., 2024). It is also significant that SA is involved in ROS signaling and that antioxidants modulate it. Plants’ regular aerobic metabolism often produces and scavenges a variety of ROS, including O^--2^, H_2_O_2_, and -OH in the process. Specifically, low ROS levels may be able to induce and/or regulate plant responses to different stress conditions and play significant signaling roles (Xu et al., 2024). Oxidative stress, a physiological condition, is the end result of an imbalance between ROS production and scavenging caused by (abiotic) stressors. Accordingly, unchecked oxidative stress can have a variety of negative effects, including oxidative alteration of essential macromolecules, cell death, and halting plant growth and development (Faizan et al., 2024).

JA was given to wheat seedlings, notable improvements in hormonal balance were seen. Both cultivars’ levels of CKs were considerably lower in salt-stressed plants during both growing seasons, particularly in the shoots. JA increased the quantity of CKs in the roots and shoots of non-stressed plants compared to control plants. JA caused the amount of CKs to recover to a higher level than in similarly stressed plants that were not treated with JA, in addition to raising the amount of CKs in stressed plants to higher levels than in non-stressed plants. Tolerance mechanisms that JA triggered in salt-stressed plants decreased Na^+^ uptake and transport to leaf organs to levels comparable to those of non-stressed plants. Thus, in both wheat cultivars, JA triggered the exclusion mechanism under salinity stress, which led to a decrease in Na^+^ levels. In both wheat cultivars, the primary regulatory function for a number of biochemical pathways may be the decrease in ion toxicity brought about by exogenous JA treatment. By decreasing Na^+^ uptake in the root and inducing salt tolerance in maize genotypes, Shahzad et al. (2015) observed Na^+^ exclusion by JA. Since K^+^ is a common metabolic characteristic under salinity stress and Na^+^ has an antagonistic effect on it, Na^+^ buildup found in many plant organs under salt stress is typically linked to a large drop in K^+^ level (Javed et al., 2021). As a result, saline environments cause delays in K^+^-related metabolic activities like Gs, WUE, and photosynthetic efficiency (Al-harthi et al., 2021). Significant variations in Na^+^ or K^+^ uptake in plant organs other than leaves were found in the investigations, whether they were cultivars, treatments, or their interactions. Seasonal variations in the genotypes’ reactions to JA and salinity are demonstrated by the highly significant Y × C × T interaction.

The main way that exogenous JA treatment to plants under salt stress decreased ion toxicity and its effects was by preserving Na^+^ and K^+^ homeostasis. Applying JA topically to strawberry and summer squash seedlings under salt stress conveniently raised their K^+^ level (Al-harthi et al., 2021). To deal with salt stress, plants deploy intricate ion transporters. The leaves, sheaths, shoots, and roots of two wheat cultivars treated with salt showed elevated relative expression levels of genes (SOS1, NHX2, and HVP1) involved in Na^+^ absorption, transport, and sequestration. In response to salt stress, SOS1 transcript levels were lowest in roots and greatest in leaves; for NHX2, the opposite pattern was seen. Sequestration of Na^+^ into vacuoles may be a key component of wheat’s salt tolerance mechanism, as indicated by the reaction that accompanied the largest increase in the percentage of Na^+^ ions in leaves and the smallest increase in Na^+^ in roots. Elevated ABA levels can explain this reaction by increasing the transport and accumulation of Na^+^ in leaves and reducing its exclusion in roots (Cabot et al., 2009). Additionally, ABA increases the expression of salt-resistant genes for Na^+^/H^+^ antiporters, HVP1 and HVP10, and vacuolar H^+^-pyrophosphatases (Yu et al., 2007). The NHX2 gene’s primary function is to sequester Na^+^ within the vacuole. The higher amount of HVP1 present in saline plants may be the reason why plants under salt stress show increased WUE (Haq et al., 2019). The process of dangerous salts being sequestered into vacuoles has been demonstrated to be initiated by HVP1 acting as a potent pump.

Jasmonic acid–SA crosstalk constitutes a critical regulatory network that modulates plant responses to salinity stress by integrating defense signaling with growth and metabolic adjustments. Although JA and SA pathways have traditionally been viewed as antagonistic—where SA predominantly mediates resistance against biotrophic pathogens and JA is associated with necrotrophic defense and abiotic stress tolerance—recent evidence suggests that their interaction is highly dynamic and context-dependent. At the molecular level, this crosstalk is governed by key regulators such as NON-EXPRESSOR OF PATHOGENESIS-RELATED GENES 1 (NPR1), MYC2, and TGA transcription factors, which coordinate the balance between JA- and SA-responsive gene expression. Under salinity stress, JA–SA interplay plays a pivotal role in maintaining reactive oxygen species (ROS) homeostasis by modulating antioxidant defense systems, thereby limiting oxidative damage to cellular components (Bali et al., 2019). Furthermore, this hormonal interaction contributes to the regulation of ion transport and osmotic balance, particularly by influencing Na^+^/K^+^ homeostasis and stress-responsive gene networks (Wasternack and Strnad, 2019). Importantly, emerging studies indicate that JA and SA can also act synergistically under certain stress conditions, co-activating defense-related genes, secondary metabolite biosynthesis, and osmoprotectant accumulation to enhance plant resilience (Pieterse et al., 2012). Therefore, a deeper mechanistic understanding of JA–SA crosstalk is essential for elucidating integrated hormonal signaling networks and for developing targeted strategies to improve crop tolerance to salinity stress.

## 6 Physiological processes controlled by jasmonic acid and salicylic acid during salinity stress

**Figure 4:**
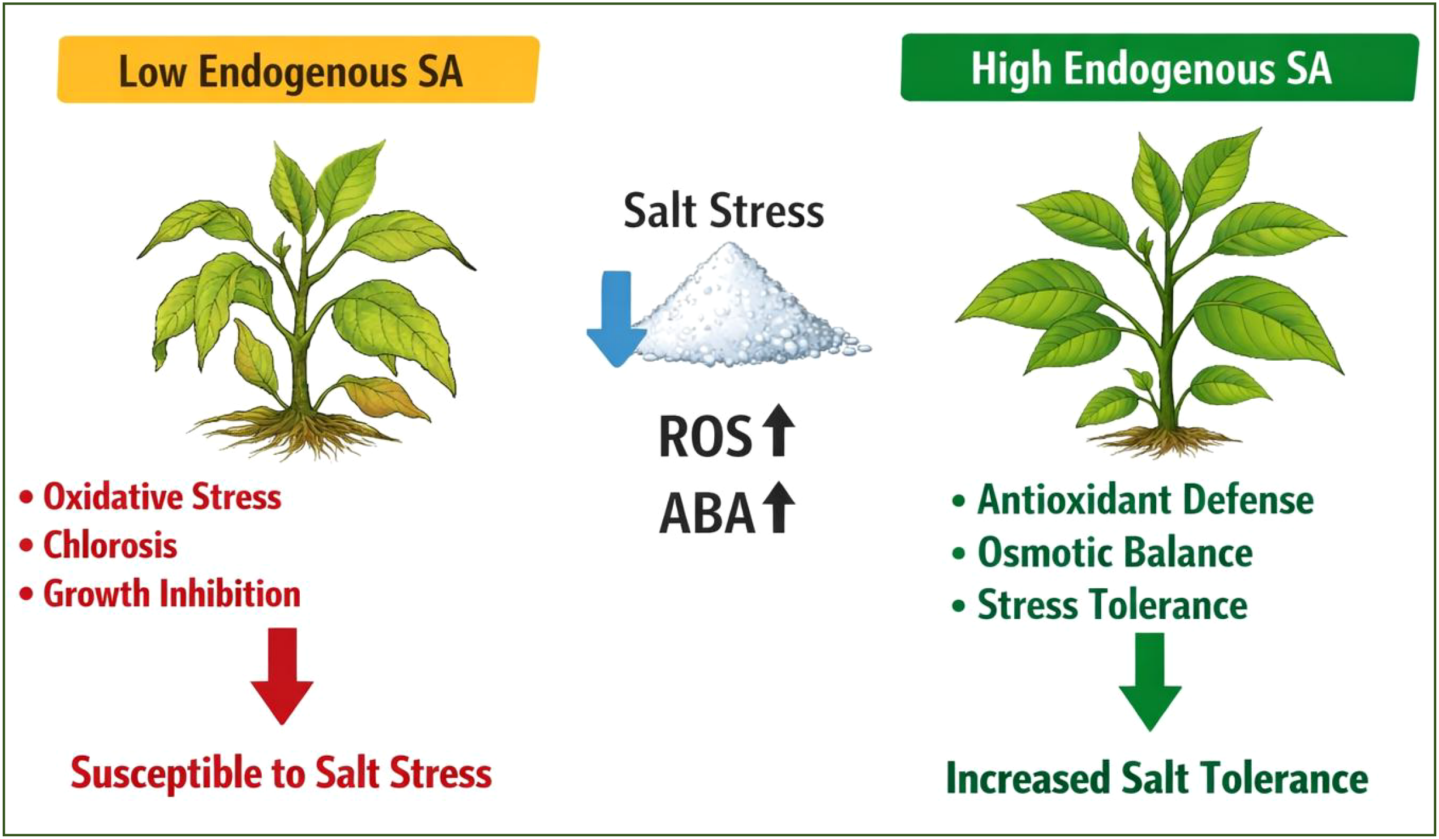
Effects of salt stress in reaction to the endogenous SA level.

Photosynthesis is frequently suppressed under abiotic stress situations, which affects crop production and carbon assimilation. SA has been shown to improve photosynthetic efficiency by increasing chlorophyll content, maintaining stomatal conductance, and protecting the photosynthetic machinery from stress-induced damage (Angon et al., 2024). SA’s impact on gene expression, antioxidant defense, osmotic control, photosynthesis, and hormonal cross-talk all support its function in abiotic stress tolerance. SA’s capacity to mediate these reactions demonstrates how effective it can be in boosting crop resilience to environmental stressors. These findings imply that SA synthesis and accumulation are critical for seed germination, especially when salt stress is present. But in barley (Xie et al., 2007), maize (Guan and Scandalios, 1995), and Arabidopsis (Lee et al., 2010), SA inhibited seed germination in a dose-dependent manner. It seems that the SA concentrations utilised in the aforementioned research are to blame for the aforementioned disparities. For instance, exogenous administration of less than 50 μM SA reduced the inhibitory effect of salt stress on sid2 mutant germination, whereas exogenous administration of more than 100 μM SA increased inhibition (Lee et al., 2010). Furthermore, a proteomic study employing transgenic plants lacking SA showed that the germination of NahG plants deficient in SA was markedly slowed down in high salinity; however, exogenous SA treatment reversed this delayed germination (Rajjou et al., 2006). Nevertheless, according to previous research, salt stress had no effect on NahG germination (Lee et al., 2010). The balance of ROS is one way that SA controls germination under salt stress (Lee et al., 2010). Due to the “self-reinforcing feedback loop” that SA and H_2_O_2_ produce in reaction to various abiotic stressors, where SA raises H_2_O_2_ concentration and H_2_O_2_ starts SA accumulation, this could be the case (Rao and Davis, 1999). The concentration and kind of plant determine how exogenous SA affects growth. In many plant species, SA improved growth at relatively low concentrations (less than 100 μM) while inhibiting growth at relatively high concentrations (more than 1 mM) (Rivas-San Vicente and Plasencia, 2011). These effects have been linked to alterations in stomatal conductance, photosynthesis, and transpiration (Li et al., 2025), as well as other hormones (Shakirova et al., 2003). Characterisation of Arabidopsis mutants with changed SA accumulation made it abundantly evident how important SA is for plant growth (Rivas-San Vicente and Plasencia, 2011). One explanation for the aforementioned growth disparities is that SA negatively regulates cell division and growth. Under salt stress, no observable developmental patterns were seen in mutants with varying SA concentrations. While SA-deficient plants (NahG, sid2, and eds5) exhibited a significant decline in growth under salt stress, SA hyperaccumulating mutants, specifically siz1 (small ubiquitin-like modifier E3 ligase1) and aba3-1 (ABA biosynthesis mutant 3-1), did not exhibit any change in growth (Miura et al., 2011; Asensi-Fabado and Munné-Bosch, 2011). Furthermore, the development of the NahG siz1 double mutant was delayed during salt stress, whereas NahG growth was increased (Hao et al., 2012; Miura et al., 2011). Due to the concentration-dependent effect of exogenous SA on photosynthesis, further research is necessary to ascertain the precise role of SA in plant growth under salt stress (Ashraf et al., 2010). At low concentrations (less than 10 μM), SA counteracted the reduction in photosynthesis caused by salt in many plant species by increasing photosynthetic rate (Li et al., 2025; Nazar et al., 2011), carbon fixation, transpiration, stomatal conductance (Li et al., 2025), and antioxidant activity (Szepesi et al., 2008). High doses of SA (1–5 mM) produced contradictory results (Nazar et al., 2011). Indeed, millimolar concentrations of SA reduced the net photosynthetic rate (Nemeth et al., 2002), inhibited the production of Rubisco hesis (Pancheva and Popova, 1997), increased the volume of chloroplasts, and decreased the amount of chlorophyll (Moharekar et al., 2003). Rubio hesis (Pancheva and Popova, 1997), decreased chlorophyll concentration (Moharekar et al., 2003), and increased chloroplast volume are the causes of stroma coagulation and swelling of thylakoid grana (Uzunova and Popova, 2000). Characterising Arabidopsis plants with changed endogenous SA concentrations revealed no discernible trends. Compared to SA-overaccumulating SA-deficient NahG had larger levels of chlorophyll and a variable-to-maximum fluorescence ratio (FV/Fm), which is a sign of PSII damage (Hao et al., 2012). Chlorophyll content and the Fv/Fm ratio did not significantly differ between hyperaccumulating (aba3) and SA-deficient (sid2 and eds5) Arabidopsis mutants under salt stress (Asensi-Fabado and Munné-Bosch, 2011). Additionally, more research is required to completely understand how SA affects photosynthetic metrics under salt stress. The mechanisms behind the preservation of photosynthetic capability depend heavily on stomata. Toxin tolerance, transpiration, and photosynthetic capacity are all impacted by stomatal opening and closure. The phytohormone abscisic acid (ABA) is known to be necessary for stomatal closure as well as drought and water scarcity resistance. Through NADPH oxidase’s generation of ROS species, ABA influences stomatal closure (Acharya and Assmann, 2009). SA stopped ABA from closing stomata. According to Rivas-San Vicente and Plasencia (2011), 0.4 mM SA reduced stomatal gas exchange by four times in just two hours by causing stomatal closure in Arabidopsis. Notably, the Arabidopsis wrky54wrky70 mutant demonstrated resistance to osmotic stress induced by PEG, which was linked to better water retention and stomatal closure. High levels of endogenous SA are known to accumulate in this mutant. ROS production via a reaction catalysed by peroxidase rather than NADPH oxidase is another mechanism by which SA-induced stomatal closure takes place. Remarkably, a siz1 Arabidopsis mutant exhibiting a higher endogenous SA concentration exhibited a smaller stomatal aperture and greater salt resistance (Miura et al., 2011). Additionally, the role that SA plays in altering specific ion transporters in roots under salt stress has been overlooked. At salt stress, membrane transporters thus control K^+^ homeostasis, Na^+^ uptake, and Na^+^ redistribution (Hayat et al., 2010) (**Figure 4**).

Salinity has an adverse effect on water levels and leaf expansion. Moreover, turgor reduction and stomatal closure brought on by an imbalance in plant water status stop growth by reducing photosynthesis (Elhamahmy et al., 2021; Elsheery et al., 2020). Salt stress conditions were used to grow broccoli and cauliflower seeds, and plants treated with salt exhibited changes in seed physiological activity. Furthermore, under saline stress, germination of seeds is impacted by water levels, amino acid content, and nutrient reserve (Arif et al., 2020). Furthermore, under salt stress, a number of plant species displayed noticeably lower growth parameters (For example, Arabidopsis showed a decrease in phosphate activity) (Nasri et al., 2016). Additionally, Balanites aegyptiacea showed decreased water leaf levels and dry weight of leaves and roots (Khamis et al., 2016). When plants were cultivated in saline conditions, there were decreases in the quantity and mass of cotton balls as well as in crop quality (Wang et al., 2018). Salinity stress also decreased tomatoes (*Solanum Lycopersicum* L.) growth parameters and leaf water potential (Xue et al., 2021; Hernández and Almansa, 2002). Different strawberry cultivars that are irrigated with 35 mM NaCl have different plant growth parameters depending on salinity. The shoots and dry roots’ weight decreased by 11–13%, 45–15%, and 29–33%, respectively, after seven days, as did the relative leaf water levels (Karlıdag et al., 2011). Surprisingly, the soil salinity varied among genotypes of the same plant species. A substantial genotype-by-genotype variation in growth parameters was observed ten days later. Genotype 14P11, for instance, had the lowest shedding rate and a significant reduction in leaf length. Additionally, the highest stomatal density values and significantly smaller epidermal cells were found in genotype 14P11. It has been observed that plants treated with salt exhibit differential modulation in stomatal expansion as opposed to epidermal cells. Genotype 6K3, on the other hand, displayed a number of traits, including the highest abscission rate and leaf necrosis. However, the genotype with the lowest leaf physiology and morphology was 2AS11, which is tolerant (Abbruzzese et al., 2009).

Physiological processes that are interrelated and under control lead to improved plant growth and development. Plant responses to abiotic stress are determined by physiological processes that are influenced by a variety of external factors (Ullah et al., 2024). Environmental factors that limit plant growth, for example, are caused by multiple physiological mechanisms. Plant biomass is increased by photosynthesis, which is regarded as the most significant physiological process. Plant development is thus negatively impacted by environmental factors that restrict photosynthesis. Additionally, salinity may have immediate or long-term impacts on the mechanism of photosynthesis. For instance, there is a short-term effect of a significant reduction in carbon uptake a few hours or one to two days after beginning treatment. A few days after treatment, salt builds up in developing leaves, which has the long-term effect of lowering carbon absorption and the net photosynthetic rate (Nazari et al., 2024). Furthermore, several studies have shown that photosynthetic efficiency is decreased by salt stress. Under conditions of salt stress, plant leaves exhibit decreased rates of respiration, CO_2_ assimilation, photosynthesis, and chlorophyll content. When Moradi and Ismail (2007) compared the tolerant cultivar, IR651, to the IR29-sensitive cultivar, they discovered that salt stress progressively reduced electron transport, CO_2_ fixation, and stomatal closure. Photosynthesis involves multiple mechanisms, including intermediates and enzymes. In conclusion, the efficiency of photosynthesis is influenced by a number of metabolic processes, including the intracellular transport of photosynthetic components, photochemical reactions, carbon absorption enzymes, and photosynthetic apparatus components (Nazari et al., 2024). In various genotypes of olive (*Olea europea* L.) treated with salt and irrigated with 200 mM brine, stomatal and mesophyll conductance were decreased in addition to photochemical efficiency (Loreto et al., 2003). The rates of photosynthesis, osmotic potential, electron transport rate, and CO_2_ assimilation in the chloroplasts of rice leaves were all reduced in plants treated with rice salt. However, because of the chloroplasts in the leaves, the rate of photosynthesis dropped. Moreover, stomatal restrictions caused by salinity reduced the osmotic potential of leaves. Under salt stress, plant leaves’ decreased mesophyll conductance was associated with ion and osmotic levels (Wang et al., 2018). On the other hand, stomatal conductance limitation lowers CO_2_ availability in the carboxylation mechanism and photosynthetic activity. Remarkably, the first stage of salinity’s detrimental effects on barley (Hordeum vulgare) cultivars’ photosynthesis is ascribed less to a decline in PSII activity and more to a restriction in stomatal conductance (Kalaji et al., 2011). In two strawberry cultivars irrigated with 35 mM NaCl, the impact of salinity on stomatal conductance has been documented. Following seven days of salt stress, readings for stomatal conductance and leaf chlorophyll decreased to 71–55% and 12–13%, respectively (Karlıdag et al., 2011). Additionally, in tomatoes cultivated at varying salt concentrations, a number of stomatal closure-related parameters suffered. Additionally, salinity encouraged the decrease of a number of stomatal characteristics, including width, length, area, perimeter, and density. This, in turn, reduced the quantity of chlorophyll and the rate at which transpiration and photosynthetic processes occurred (Kumar et al., 2024). Additionally, compared to the control, the pea salt-treated plant’s stomatal conductance dropped after 48 hours of salt treatment (Kanwal et al., 2024).

Jasmonic acid (JA) was initially identified as a hormone associated with stress responses in higher plants, but it is now recognized as an important endogenous signaling molecule that regulates plant growth and development. Since its discovery in 1962, jasmonates have been reported to play diverse roles in plant biological systems, including cell movement, stress tolerance, and reproductive development (Shekhawat et al., 2023). In nature, jasmonates are lipid-derived compounds belonging to the oxylipin family and are generally present as hormonal lipids and carboxylic acids. The term jasmonates (JAs) broadly refers to jasmonic acid and its various derivatives, including lactones of 12-hydroxy-JA-Ile, 12-O-glucosyl-JA-Ile, methyl jasmonate (MeJA), jasmonate–amino acid conjugates, cis-jasmon, and jasmonoyl-isoleucine (Alirzayeva et al., 2017).

JA is synthesized from cyclopentanone compounds that belong to the oxylipin family, which are lipid-based signaling molecules involved in plant responses to environmental stimuli. Because of this origin, jasmonates are considered an important class of lipid-derived signaling molecules that regulate plant responses to various abiotic stresses such as heavy metal toxicity, salinity, and drought. The concentration of JA varies among plant tissues, with relatively low levels found in mature roots and leaves, while significantly higher concentrations occur in flowers and other reproductive organs (Kanwar et al., 2020).

JA plays several vital physiological roles in plants, including regulation of cell division, development of reproductive structures, fruit ripening, nutrient uptake (particularly phosphorus and nitrogen), stomatal movement, electron transport processes, and carbohydrate transport (Pandey, 2018). Additionally, jasmonates are involved in pollen viability, root and stem development, and the regulation of fruit maturation processes (Meng et al., 2009). Among JA derivatives, methyl jasmonate (MeJA) has been widely studied for its role in improving fruit quality and stress responses. In climacteric fruits such as apples, the application of MeJA has been shown to enhance desirable traits by increasing phenolic compound levels, improving red coloration, and promoting the accumulation of anthocyanins and carotenoids. MeJA treatment also stimulates the production of ethylene and various ester compounds associated with fruit aroma and ripening (Ni et al., 2020).

In contrast, MeJA induces different physiological responses in non-climacteric fruits such as raspberries, blackberries, and strawberries. Moreover, jasmonates function as important signaling molecules under abiotic stress conditions, regulating the expression of stress-responsive genes and thereby enhancing plant tolerance to environmental stresses (Allagulova et al., 2020).

## 6 Jasmonic acid and salicylic acid signaling networks in plant salinity stress: transcriptional and epigenetic regulation

Salinity stress imposes severe ionic, osmotic, and oxidative constraints on plant growth and productivity. Among phytohormones, JA and SA function as central regulators of stress perception, signal transduction, and transcriptional reprogramming during salt exposure. JA is rapidly synthesized via the octadecanoid pathway through the sequential action of lipoxygenase (LOX), allene oxide synthase (AOS), and allene oxide cyclase (AOC), generating the bioactive conjugate JA-Ile, which is perceived by the SCF^COI1–JAZ co-receptor complex. Salinity induces JA accumulation, promoting the proteasomal degradation of JAZ repressors and the release of MYC2/3/4 transcription factors, which activate stress-responsive genes involved in ion transport, antioxidant defense, and secondary metabolism (Wasternack and Strnad, 2019). JA signaling enhances Na^+^ compartmentalization through the induction of HKT1, and SOS1, while simultaneously stimulating osmoprotectant biosynthesis (e.g., P5CS for proline accumulation) and antioxidant enzymes including SOD, CAT, APX, and GR (Qiu et al., 2014). At the transcriptional level, MYC2 directly regulates JA-responsive promoters containing G-box elements, coordinating ROS detoxification and ionic homeostasis. JA also interacts with ABA signaling via SnRK2–bZIP (ABF/AREB) modules, reinforcing stomatal regulation and stress-induced metabolic shifts under salinity. Salicylic acid, synthesized primarily via the isochorismate synthase pathway (SID2) and the phenylalanine ammonia-lyase (PAL) route, acts as a key modulator of redox balance and photosynthetic protection under salt stress. Under moderate salinity, SA improves K^+^ retention, stabilizes chlorophyll biosynthesis, and suppresses lipid peroxidation by strengthening the ascorbate–glutathione cycle. However, excessive SA accumulation can promote ROS overproduction and growth inhibition, highlighting the importance of precise SA homeostasis during salt adaptation (Horváth et al., 2007). JA–SA interactions under salinity are highly context dependent, displaying both antagonistic and synergistic behaviors. The transcription factor WRKY70 acts as a key molecular switch integrating JA–SA crosstalk by differentially regulating defense and stress-responsive gene sets. Under salt stress, antagonism between SA-NPR1 and JA-MYC2 pathways allows plants to balance oxidative defense and ion detoxification, whereas synergistic activation is observed at the level of shared downstream targets such as antioxidant enzymes and stress-induced chaperones. Mitogen-activated protein kinase (MAPK) cascades (MPK3, MPK6) and Ca^2+^-dependent protein kinases (CDPKs) function as upstream integrators of JA and SA biosynthesis during salinity-induced signal amplification (Zhang et al., 2018).

**Figure 5:**
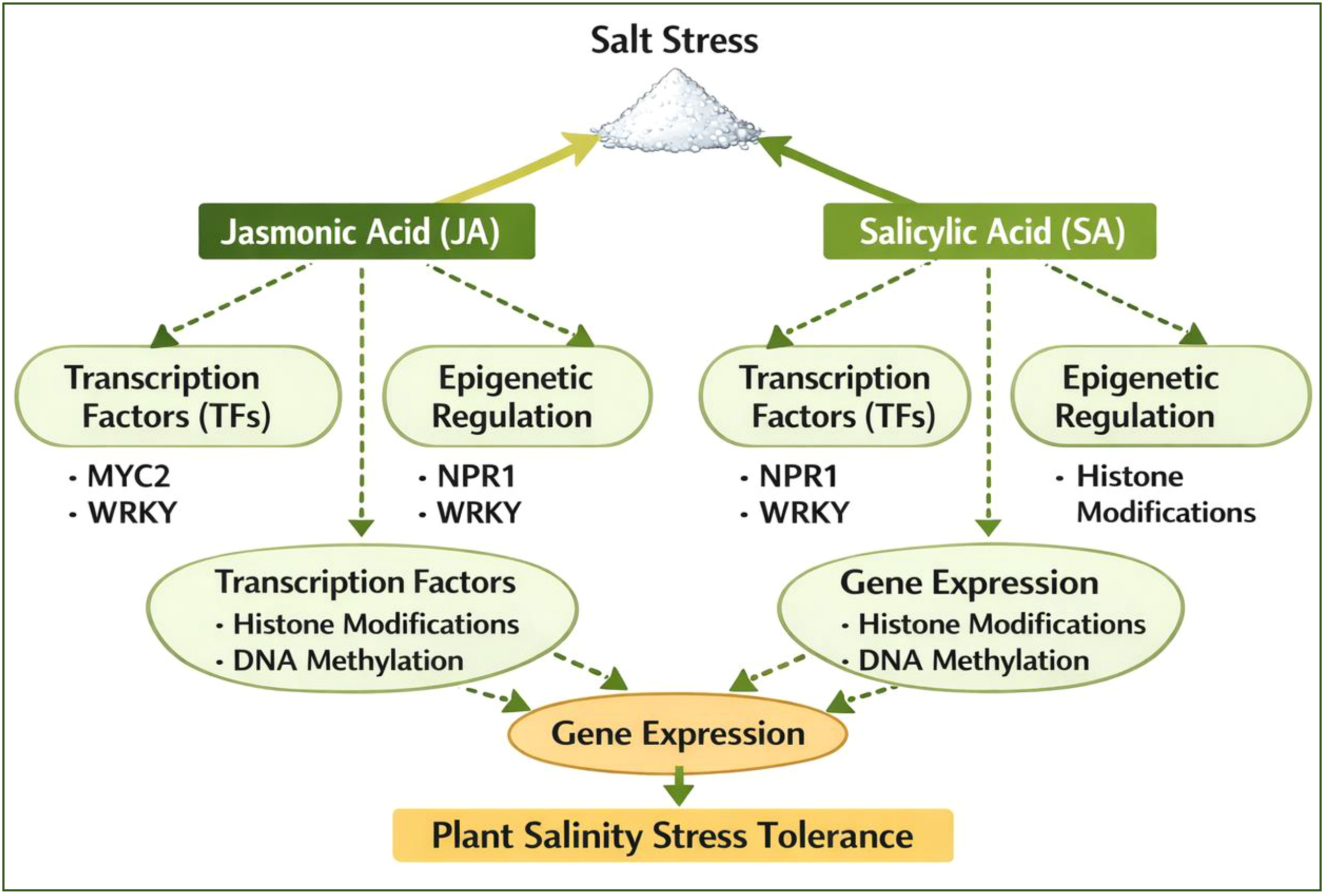
Schematic representation of JA and SA signaling networks in plant responses to salinity stress, highlighting transcriptional and epigenetic regulation.

Epigenetic regulation adds an additional layer of control to JA–SA responsiveness under salinity stress. Salt exposure induces dynamic changes in DNA methylation (MET1, CMT3, DRM2) and histone modifications, particularly H3K4me3 and H3K9ac at promoters of JA/SA-responsive genes, including AOS, WRKYs, and MYC2. Chromatin remodeling complexes such as SWI/SNF further modulate transcriptional accessibility of stress-inducible loci. These epigenetic marks contribute to stress memory, allowing faster and stronger JA–SA activation following recurrent salinity (**Table 2**) exposure, with potential transgenerational effects. At the transcription factor level, multiple TF families integrate JA–SA signaling into coordinated stress-adaptive gene networks. MYC2, NACs (NAC2), WRKYs (WRKY25, WRKY33, WRKY70), bZIPs (ABFs), and DREB/CBFs act as convergence nodes linking hormonal, ionic, and redox signaling. These TFs regulate gene clusters involved in ion transport (SOS pathway), osmolyte biosynthesis (P5CS, BADH), antioxidant defense (APX, GPX), and hormonal feedback regulation (JAZ). The coordinated activity of these regulators ensures metabolic stability and growth maintenance during prolonged salinity stress (**Figure 5**).

**Table 2:** Comparative effectiveness of jasmonate application methods in different crops under stress conditions.

Application method	Mode of application	Crops	Effects	Effectiveness under stress	References
Foliar Spray	Exogenous spraying of JA/MeJA on leaves	Soybean, rice, tomato, safflower	↑ Antioxidant enzymes (SOD, CAT, POD), ↑ chlorophyll content, ↑ photosynthesis, improved gas exchange, ↑ biomass and yield	Highly effective for rapid stress mitigation (salinity, drought, heavy metals); immediate response at vegetative and reproductive stages	Zeeshan et al. (2023)
Seed Priming	Pre-sowing soaking of seeds in JA/MeJA solution	Wheat, maize, chickpea, rice	↑ Germination rate, ↑ seedling vigor, improved osmotic adjustment, early activation of antioxidant defense and stress-responsive genes	Effective at early growth stages; enhances tolerance to salinity and drought during establishment phase	Ali et al. (2023)
Hydroponic Application	JA/MeJA added to nutrient solution in controlled systems	Tomato, *Arabidopsis thaliana*, lettuce	Precise regulation of JA uptake, ↑ root growth, improved ion homeostasis (K^+^/Na^+^ balance), reduced oxidative damage and metal toxicity	Highly effective under controlled conditions; ideal for mechanistic and physiological studies	Wasternack and Strnad (2019)
Soil Application	JA incorporated into soil or growth medium	Rice, *Brassica juncea*, maize	Improved nutrient uptake, modulation of rhizosphere activity, ↑ stress resilience, enhanced enzymatic activity	Moderately effective; provides sustained response but slower compared to foliar application	Ndiaye et al. (2022)

↑ Increase.

## 7 Conclusions and future prospects

In plants subjected to oxidative stress, salinity decreases the membrane stability index and increases MDA and H_2_O_2_ levels. Soluble sugars, proline, and glycine betaine are increased by SA and JA, which reduce oxidative stress. A complex web of reactions to environmental stressors is regulated by SA, a key plant hormone. SA functions through a complex web of interconnected mechanisms, including osmotic regulation, antioxidant defense, gene expression modulation, signaling pathways, and hormonal cross-talk. Because of this interaction, SA can both reduce stress and promote growth and development, making it a valuable tool for crop management. Thus, the molecular and biochemical processes underlying SA-mediated stress reduction must be understood. JA priming greatly enhanced all plant growth parameters and yield characteristics of plant cultivars under both saline and control conditions. Furthermore, plants under salinity stress can benefit from JA treatment by reducing the co-stress factors (osmotic stress, oxidative stress, and tissue dehydration). Since JA can improve PSII’s structural stability and functional activity, this alleviating effect can be explained. Additionally, JA improved Na^+^ transport and exclusion in different organs by up-regulating a number of important genes that may be involved in Na^+^ sequestration, transport, and uptake (e.g., SOS1, NHX2). After a subsequent exposure to salinity stress, JA seed inoculation can enhance plant tolerance to salinity stress and effectively reduce stress-sensitive criteria in an environmentally responsible manner. Phytohormones enhanced net photosynthesis and stomatal conductance even in the presence of salt stress. This study demonstrates how salinity stress dramatically lowers plant physiological traits and growth. All things considered, it demonstrated that SA shields plants from salinity by promoting plant development and strengthening their morphysiological and biochemical characteristics. Future research should focus on (i) spatiotemporal hormone dynamics at single-cell resolution, (ii) CRISPR-mediated editing of key JA–SA regulators (MYC2, WRKY70) to enhance salt tolerance without yield penalties, (iii) epigenome editing of stress-responsive loci, and (iv) multi-omics integration (transcriptome, epigenome, metabolome) to decode hormone-driven regulatory hierarchies under saline environments. Translating these advances into breeding programs will be critical for developing climate-resilient crops with sustained productivity on salt-affected soils.
